# EFFECTIVENESS OF HOME- AND COMMUNITY-BASED SERVICES IN DECREASING HEALTH CARE EXPENDITURE IN TAIWAN

**DOI:** 10.1093/geroni/igz038.570

**Published:** 2019-11-08

**Authors:** Ya-Mei Chen, Hsiao-Wei Yu, Ying-Chieh Wang

**Affiliations:** 1 National Taiwan University, Taipei, Taiwan; 2 Chang Gung University of Science and Technology, Taipei, Taiwan; 3 Institute of Health Policy and Management, Taipei, Taiwan

## Abstract

Ideally, continuum of care involves wide-ranging health and long-term care (LTC) services. Taiwan’s National Health Insurance scheme and 10-Year Long-term Care Plan attempts to provide universal and fundamental services of continuum care. However, the accessibility of these services for care recipients remains unclear. This study aims to examine the effectiveness of continuum care in decreasing the healthcare expenditure of LTC recipients using home- and community-based services (HCBS). Data collated from the 2010–2013 Long-Term Care Service Management System (N = 77,251) were subjected to latent class analysis to identify subgroups of recipients using HCBS. Subsequently, the 1-year primary care expenditure after receiving HCBS was compared through generalized linear modeling. Three discrete HCBS subgroups were found: home-based personal care (HP), home-based health care (HH), and community-based care (CC). No difference in the number of visits to doctors and the average primary care expenses was observed between the HP and HH subgroups. However, considering physical and psychosocial confounders, care recipients in the CC subgroup recorded a higher number of visits to doctors (β = 3.05, SD = 0.25, p < 0.05) and lower primary care expenditure (β = -98.15, SD = 43.17, p = 0.02) than the other two subgroups. These findings suggest that LTC recipients in Taiwan may obtain better continuum care only for CC service recipients. Additionally, community-based LTC services may lower the cost of health expenditure after 1 year.

